# Mineralocorticoid Antagonist Improves Glucocorticoid Receptor Signaling and Dexamethasone Analgesia in an Animal Model of Low Back Pain

**DOI:** 10.3389/fncel.2018.00453

**Published:** 2018-11-22

**Authors:** Shaimaa I. A. Ibrahim, Wenrui Xie, Judith A. Strong, Raquel Tonello, Temugin Berta, Jun-Ming Zhang

**Affiliations:** ^1^Pain Research Center, Department of Anesthesiology, University of Cincinnati College of Medicine, Cincinnati, OH, United States; ^2^Graduate Program in Molecular, Cellular, and Biochemical Pharmacology, University of Cincinnati College of Medicine, Cincinnati, OH, United States

**Keywords:** back pain, epidural steroids, inflammation, glucocorticoids, dorsal root ganglion, mineralocorticoid receptor, glucocorticoid receptor

## Abstract

Low back pain, a leading cause of disability, is commonly treated by epidural steroid injections that target the anti-inflammatory glucocorticoid receptor (GR). However, their efficacy has been controversial. All currently used epidural steroids also activate the pro-inflammatory mineralocorticoid receptor (MR) with significant potency. Local inflammation of the dorsal root ganglia (DRG), a rat model of low back pain, was used. This model causes static and dynamic mechanical allodynia, cold allodynia and guarding behavior (a measure of spontaneous pain), and activates the MR, with pro-nociceptive effects. In this study, effects of local Dexamethasone (DEX; a glucocorticoid used in epidural injections), and eplerenone (EPL; a second generation, more selective MR antagonist) applied to the DRG at the time of inflammation were examined. Mechanical and spontaneous pain behaviors were more effectively reduced by the combination of DEX and EPL than by either alone. The combination of steroids was particularly more effective than DEX alone or the model alone (3-fold improvement for mechanical allodynia) at later times (day 14). Immunohistochemical analysis of the GR in the DRG showed that the receptor was expressed in neurons of all size classes, and in non-neuronal cells including satellite glia. The GR immunoreactivity was downregulated by DRG inflammation (48%) starting on day 1, consistent with the reduction of GR (57%) observed by Western blot, when compared to control animals. On day 14, the combination of DEX and EPL resulted in rescue of GR immunoreactivity that was not seen with DEX alone, and was more effective in reducing a marker for satellite glia activation/neuroinflammation. The results suggest that EPL may enhance the effectiveness of clinically used epidural steroid injections, in part by enhancing the availability of the GR. Thus, the glucocorticoid-mineralocorticoid interactions may limit the effectiveness of epidural steroids through the regulation of the GR in the DRG.

## Introduction

Low back pain is a major health issue and common cause of chronic pain. It is prevalent in one third of the US population (Johannes et al., 2010; Ibrahim et al., 2016). It is the most common cause of chronic pain. It is costly due to reduced worker productivity, lost wages and medical treatment and is a leading cause of disability in US and worldwide (Johannes et al., 2010; Institute of Medicine (US) Committee on Advancing Pain Research, Care, and Education, 2011; Toblin et al., 2011; Gaskin and Richard, 2012). Low back pain can be caused by conditions such as degenerative disc diseases (Ross, 2006; Kallewaard et al., 2010) and compression of nerve roots (Qu et al., 2016). Inflammation is a major player in all these conditions (Finnerup et al., 2010; Institute of Medicine (US) Committee on Advancing Pain Research, Care, and Education, 2011; Toblin et al., 2011). Epidural steroid injection has become a standard treatment for low back pain management, to reduce pain and inflammation in the aforementioned conditions. However, adequate treatment was not reported in many cases (Institute of Medicine (US) Committee on Advancing Pain Research, Care, and Education, 2011; Benoist et al., 2012; Manchikanti et al., 2012; Andersson, 2014; Manchikanti et al., 2014; Shamliyan et al., 2014). Mixed clinical trial results range between short-term and long-term benefits to no benefits at all (Benoist et al., 2012; Manchikanti et al., 2012; Meng et al., 2015). Dexamethasone (DEX), a high potency, long acting glucocorticoid has been increasingly used as an epidural steroid injection for low back pain management, in part because it is viewed as a safer, non-particulate steroid (Schneider et al., 2015). Other steroids commonly used for epidural injections include betamethasone, 6-α-methylprednisolone and triamcinolone (Ibrahim et al., 2016). These clinically used steroids activate the glucocorticoid receptor (GR). However, they also activate the mineralocorticoid receptor (MR) *in vitro* with significant potency (Grossmann et al., 2004; Sedlák et al., 2011). MR is expressed in cells other than kidney such as cardiomyocytes (Messaoudi and Jaisser, 2011), brain neurons (Joels et al., 2008) and dorsal root ganglia (DRG) neurons (Dong et al., 2012). In other tissues, MR activation is pro-inflammatory and implicated in organ damage such as in heart, kidney and vasculature (Ibrahim et al., 2016; Belden et al., 2017). The pro-inflammatory effects of MR activation can counteract the desired GR anti-inflammatory effects of the epidural steroid injection. Therefore, it may be beneficial to select the steroid with minimal MR affinity to maximize the anti-inflammatory effects.

Previously, we have demonstrated that MR is expressed in the DRG, and that it translocates to the nucleus 1 day after inflammation. In addition, an MR antagonist eplerenone (EPL) combined with 6-α-methylprednisolone improved its efficacy (Ye et al., 2014). In this study, we used an animal model of low back pain, local inflammation of the DRG (LID), to mimic clinical low back pain conditions. This model involves a local injection of the immune stimulator zymosan in the vicinity of the L5 DRG (Xie et al., 2006). We examined the effects of DEX, which is used clinically for epidural steroid injections, and the MR antagonist EPL, which is clinically approved for conditions other than low back pain, such as hypertension and heart failure. We also investigated how GR immunoreactivity and neuroinflammation changed in the DRG in response to DRG inflammation and to local injections of these two steroids.

## Materials and Methods

### Animals

All surgical procedures and the experimental protocol were approved by the University of Cincinnati institutional animal care and use committee and adhered to the guidelines of the Guide for the Care and Use of Laboratory Animals. Adult Sprague-Dawley rats (8 weeks old) were purchased from Envigo (Indianapolis, IN, USA). Male and female rats were used in equal numbers in the experiments. Rats were housed two per cage in a specific pathogen free facility under a controlled diurnal cycle of 14-h light and 10-h dark with corncob bedding and free access to water and food. The ambient environment was maintained at constant temperature (22° ± 0.5°C) and relative humidity (60%–70%). Rats were acclimated to the environment and behavioral tests prior the implementation of the animal model.

### Surgical Procedure for Localized Inflammation of the DRG (LID)

The surgery was performed as previously described (Xie et al., 2012b). Briefly, rats were anesthetized with isoflurane and an incision was made on the back to expose the L5 and L4 transverse processes. The L5 DRG was inflamed by the local injection of the immune activator zymosan (Sigma-Aldrich, St. Louis, MO, USA, catalog #Z4250; 2 mg/ml, 10 μl in volume, in incomplete Freund’s adjuvant-IFA). For experiments in which DRG tissue was isolated for obtaining mRNA or protein, both L4 and L5 were inflamed.

### Local Drug Application to the Inflamed DRG *in vivo*

All steroids used were insoluble in water. Therefore, they were all added to the oily IFA/zymosan. Animals were assigned randomly to the following experimental groups were used: (1) LID group, in which the zymosan in IFA was injected locally to the DRG to create the LID model. (2) LID + EPL group, in which 500 μg EPL (Tocris, Bristol, United Kingdom, catalog #2397), a specific MR antagonist, was added to the IFA + zymosan which was used to inflame the L5 DRG (Dong et al., 2012). (3) LID + DEX group, in which DEX (30 μg, Tocris, Bristol, United Kingdom, catalog #1126), a clinically used GR agonist, was added to the IFA + zymosan. (4) LID + DEX + EPL group, in which both DEX and EPL were added to the zymosan + IFA. This method and concentrations were selected based on our previously published studies (Dong et al., 2012; Ye et al., 2014) and were originally based on a published study (Shepard et al., 2003), in which steroid micropellets implanted into the brain had a diffusion radius of ~750 μm and duration of action of 5–7 days. Using this method, aldosterone pellets in brain had behavioral effects at 15 and 30 μg but not at 3 μg (Myers and Greenwood-Van Meerveld, 2010); however, we chose to use 500 μg EPL in our study because EPL has relatively low affinity for the MR (IC_50_ several of orders of magnitude higher than the EC_50_ for aldosterone or corticosterone (Sica, 2005; Fagart et al., 2010), even though it is specific since *in vitro*, essentially no inhibition of the GR by EPL is observed (i.e., >1,000-fold higher IC_50_ for GR than for MR; Sica, 2005).

### Behavioral Assessment

Animals were assessed for pain behaviors for two trials to determine the baseline before implementing the LID pain model. The baseline values were averaged and plotted as day 0. The pain behavior was assessed as indicated at postoperative day (POD) 1, 3, 7 and 14. All behavioral testing was conducted in a dedicated room within the laboratory, starting around 9:00 A.M (i.e., ~3–4 h after lights on). Pain behaviors were tested in both contralateral and ipsilateral paws, but only the ipsilateral data is presented because there was little contralateral effect of the LID model (Xie et al., 2006). For all behavioral experiments, the tester was blinded to the experimental groups. Behavior data presented in each figure were obtained in side-by-side measurements.

#### Static Allodynia

The von Frey test measures static mechanical allodynia (i.e., responses to punctate mechanical stimuli that are normally innocuous). The thresholds for mechanical allodynia were measured with a series of von Frey filaments (Stoelting, Wood Dale, IL, USA) ranging from 0.41 g to 15.0 g. Rats were placed in an individual chamber with a plastic mesh floor and allowed to acclimate 20 min before testing. The stimuli were applied to the heel region of the paw. A cutoff value of 15 g was used, which did not usually evoke a response in naïve animals. The paw withdrawal threshold (PWT) was calculated using the up-down method (Chaplan et al., 1994).

#### Dynamic Allodynia

The cotton wisp test measures dynamic mechanical allodynia (i.e., responses to light moving mechanical stimuli that are normally innocuous; Zhang et al., 2000). A light cotton wisp was stroked mediolaterally across the plantar surface of the hind paw. Then, the presence or absence of a brisk withdrawal response was recorded and allodynia was scored as percentage of brisk withdrawals (out of six trials). This stimulus failed to evoke a response in normal animals.

#### Cold Allodynia

Acetone test was used to measure cold allodynia. Fifty microliter of acetone was applied to the ventral surface of the hind paw. Cold sensitivity was measured as percentage of withdrawal responses (out of six trials). Responses to acetone or the light brush stroke included withdrawal, several rapid paw flicks, licking, or shaking of the paw. Walking movements were not scored as positive responses.

#### Spontaneous Pain

The guarding score measures spontaneous pain, the ongoing pain present without applying a stimulus (Xu and Brennan, 2010). The paws were closely observed for six times every 5 min. The guarding score was based on the position of the paw during the majority of time during the 1 min observation period. The guarding behavior was scored as 0 (no guarding, paw flat on floor), 1 (mild shift of weight away from paw), 2 (unequal weight bearing and some part of the foot not touching the floor), or 3 (foot totally raised or not bearing any weight). These scores were recorded just before each application of the von Frey filament (6 per paw total), averaged, and presented as the spontaneous pain score.

### Immunohistochemistry (IHC)

As described previously (Dong et al., 2012), rats were anesthetized with pentobarbital sodium (40 mg/kg, intraperitoneal) and perfused through the left ventricle of the heart with 0.1 M phosphate buffer until clear fluid was seen, followed by perfusion with 4% paraformaldehyde for 20 min for fixation of the tissue. Inflamed or normal DRGs were removed and post-fixed in 4% paraformaldehyde for 1 h at room temperature, then transferred to 4% sucrose overnight at 4°C. DRGs were embedded in Tissue-Tek^®^ (O.C.T. Compound, Sakura^®^ Finetek, PA, USA). Frozen sections were cut at 10 μm on a cryostat (Leica CM1860, CA, USA) from the harvested DRGs. The sections for IHC were selected randomly for the analysis. To reduce variability, sections from all experimental groups in a given experiment were mounted side-by-side on each slide and processed together. Sacrifice of animals for IHC was always conducted starting around 9:00 A.M.

The sections were permeabilized twice for 5 min in phosphate-buffered saline with 0.3% Triton X-100 (Sigma, St. Louis, MO, USA; PBST), blocked for 1.5 h with 10% normal goat serum in PBST, and incubated overnight at 4°C with primary antibodies. After washing in PBST, sections were incubated for 2 h at room temperature with secondary antibodies conjugated with fluoro-Alexa-488 (goat anti-rabbit; 1:1,000; catalog A-11034, Invitrogen, Grand Island, NY, USA), or conjugated with fluoro-Alexa-594 (goat anti-mouse; 1:1,000; catalog A11032, Invitrogen, Grand Island, NY, USA) dissolved in 3% normal goat serum in PBST. The following primary antibodies were used: GR (1:200, catalog number sc-1004, Santa Cruz, rabbit polyclonal antibody used to identify the activated GR, also known as M-20 (Sarabdjitsingh et al., 2010) as previously used for neuronal staining (McKlveen et al., 2013); Iba-1 (1:200, goat polyclonal anti-Iba1 antibody, catalog ab5076, Abcam, used to identify macrophages), glutamine synthetase (GS; 1:200, GS, catalog ab64613, Abcam, a mouse monoclonal antibody used to identify the satellite glia), S100B (1:200, catalog SA-12, Novus, a mouse monoclonal antibody used to detect Schwann cells), Glial Fibrillary Acid Protein (GFAP; rabbit polyclonal anti-GFAP antibody, catalog 22522, RRID:AB_572240, ImmunoStar, used to identify activated satellite glia in the DRG). After drying, the sections were mounted on coverslips with Vector Hard Set mounting medium (Vector Laboratories, Burlingame, CA, USA). Control experiments included the removal of the primary or the secondary antibody. Images from multiple sections of each DRG, selected at random without regard to the amount of signal observed, were captured using an Olympus BX61 fluorescent microscope with SlideBook Digital Microscopy 6.1 Imaging acquisition Software (Intelligent Imaging Innovation, Santa Monica, CA, USA), or Olympus BX63F with CellSens Dimensions software. Overall intensity was measured and normalized by the area measured. Areas dominated by neuronal cell bodies were analyzed, rather than predominately axonal regions, unless otherwise indicated. Data from multiple sections was summarized as animal averages and the statistical analysis was applied to these average values. For immunoreactive intensity quantification, intensity/area in treated groups was calculated and normalized to that seen in normal DRG measured side-by-side. For DRG neuronal cell size profiling, Nissl stain was used to allow for the visualization of neurons. CellSens Dimensions software was used to draw a border around each neuron in selected cellular regions of the DRG, which were scored as GR-positive or GR-negative. Then, the area for each cell was exported.

### Quantitative Real-Time RT-PCR (qPCR)

Total RNA was isolated from DRGs using the Direct-zol RNA MiniPrep RNA isolation kit (Zymo Research, catalog R2050). The RNA was reverse transcribed using the High Capacity RNA-to cDNA Kit (Thermo-Fisher, catalog 4387406) by incubation at 37°C for 60 min, 95°C for 5 min, followed by a hold at 4°C. The first strand cDNA reaction was diluted 20 times in 10 mM TRIS buffer and stored at −20°C until further analysis. Quantitative real-time RT-PCR (qPCR) was performed on a QuantStudio™ 3 Real-Time PCR System (Thermo Fisher). Each 20 μl reaction included 10 μL FastStart Universal SYBR Green Master (Roche, catalog 04913850001), 1 μl (5 μM) of each forward and reverse primer, 4 μL of the cDNA template and 5 μL RNase-free water. The thermocycler protocol was: activation of the Taq polymerase at 95°C for 10 s followed by 40 cycles of denaturing at 95°C for 15 s annealing at 60°C for 1 min, extension at 76°C for 15 s followed by a DNA melting curve for the determination of amplicon specificity. Primer sequences were: Hypoxanthine Phosphoribosyl transferase (HPRT; NM_012583): GCAGACTTTGCTTTCCTTGG (forward), TACTGGCCACATCAACAGGA (reverse); GR (Nr3c1, NM_012576.2): AGCCTGACTTCCTTGGGGGCT (forward), AGCTTGGGAGGTGGTCCCGT (reverse); MR (Nr3c2, NM_013131.1): GGAGAAGTGATGGGTATCCCGTCC (forward), ACCCCATAGTGACACCCAGAAGCC (reverse). Gene expression was normalized by the housekeeping gene HPRT from the same sample. The relative expression ratio per condition was calculated based on the method described by Pfaffl (2001).

### Western Blotting

Rats were terminally anesthetized and L4-L5 DRG tissues were collected. Tissues were homogenized in RIPA lysis buffer (Sigma, catalog #20-188) with MS-SAFE protease and phosphatase inhibitor cocktail (Sigma, catalog #MSSAFE-5VL). After homogenization, 40 μg of sample was loaded per lane and proteins were separated by Bolt 4%–12% Bis-Tris Plus (Thermo Fisher Scientific, catalog #NW04125BOX) gel electrophoresis. Proteins were then transferred to PDVF membranes (Sigma, catalog #IPFL07810), which were blocked with 5% nonfat dry milk (Cell Signaling, catalog #9999) in Tris buffered saline with Tween^®^ 20 (TBST, Cell Signaling, catalog #9997) for 1 h at room temperature. Membranes were incubated overnight at 4°C with the same GR primary antibody as used for IHC (1/1,000) and mouse anti-glyceraldehyde 3 phosphate dehydrogenase (GAPDH; 1/2,000, Thermo Fisher Scientific, catalog # MA5-15738-HRP) used as a loading control, diluted with 2% BSA (Sigma, catalog #A7030) in TBST. The membranes were then washed with TBST and GR was probed with anti-rabbit secondary antibody conjugated with HRP (1/2,000, Cell Signaling, catalog #7074) for 1 h at room temperature. After washing with TBST, protein bands were visualized by enhanced chemiluminescence (Thermo Fisher Scientific, catalog #34075) and scanned with the iBright FL1000 Imaging System (Thermo Fisher Scientific). Bands were quantified using ImageJ (NIH) and GR expression normalized by GAPDH.

### Procedure for *in vivo* Injection of siRNA Into the DRG

siRNAs directed against GR and non-targeting control were designed by and purchased from Dharmacon/ThermoFisher (Lafayette, CO, USA). The GR-siRNA was siGENOME™ siRNA consisting of a “smartpool” of four different siRNA constructs combined into one reagent. Catalog numbers (Dharmacon) were M-089504-01-0005 siGENOME (directed against GR) and D-001210-02 (non-targeting control directed against firefly luciferase, screened to have minimal off-target effects and least four mismatches with all known human, mouse and rat genes according to the manufacturer). The sequences for the GR siRNA were: (construct 1) UAGCUGAAAUCAUCACUAA, (2) GACCAUGAGUAUUGAAUUC, (3) GAAAUGGGCAAAGGCGAUA, and (4) GAAAUGGGCAAAGGCGAUA. These target regions of the mRNA that are distinct from the region encoding the antigen used for generating the M-20 antibody. The luciferase sequence (UAAGGCUAUGAAGAGAUAC) is unrelated to the shRNA sequence recently reported to have extensive off-target effects in hippocampal neurons (Hasegawa et al., 2017). To demonstrate the specificity of the GR antibody, 3 μl aliquots containing 80 pmoles of siRNA made up with cationic linear polyethylenimine (PEI)-based transfection reagent (“*in vivo* JetPEI,” Polyplus Transfection, distributed by WVR Scientific, USA) at a nitrogen/phosphorus ratio of 8 were injected into L4 and L5 DRG on one side, through a small glass needle (75 μm o.d.) inserted close to the DRG through a small hole cut into the overlying membrane close to the site where the dorsal ramus exits the spinal nerve, as previously described (Xie et al., 2012a). The injection of the siRNA directed against GR or the non-targeting control was performed in normal (non-inflamed) animals, and DRG samples for IHC were obtained 4 days later.

### Data Analysis

No animals were excluded from analysis. Sample sizes were based on our previous experience with the procedures used. In general, males and females were used in equal numbers in each group and their data combined because we did not see any obvious sex differences in our previous studies using this model (Xie et al., 2016) or in the present study.

Graphpad Prism (La Jolla, CA, USA), version 6 software was used for statistical analysis. Behavioral time course data were analyzed using two-way repeated measures ANOVA with Holm-Sidak multiple comparisons posttest to determine on which days experimental groups differed. For these analyses, *F* values for the comparison between groups are given as *F*_(degrees freedom in numerator, degrees freedom in denominator)_. Except where indicated, comparisons between groups in other experiments were performed with one-way ANOVA, followed by Bonferroni’s *post hoc* analysis, or with unpaired Student’s *t*-test. Two-tailed tests were used throughout. Significance was ascribed for *p* < 0.05. Levels of significance are indicated by the number of symbols, e.g., *p = 0.01 to < 0.05; **p = 0.001–0.01; ***p < 0.001. Data are presented as Mean ± SEM except where indicated.

## Results

### EPL Enhances the Analgesic Effect of DEX After Local DRG Inflammation

**Figure 1:**
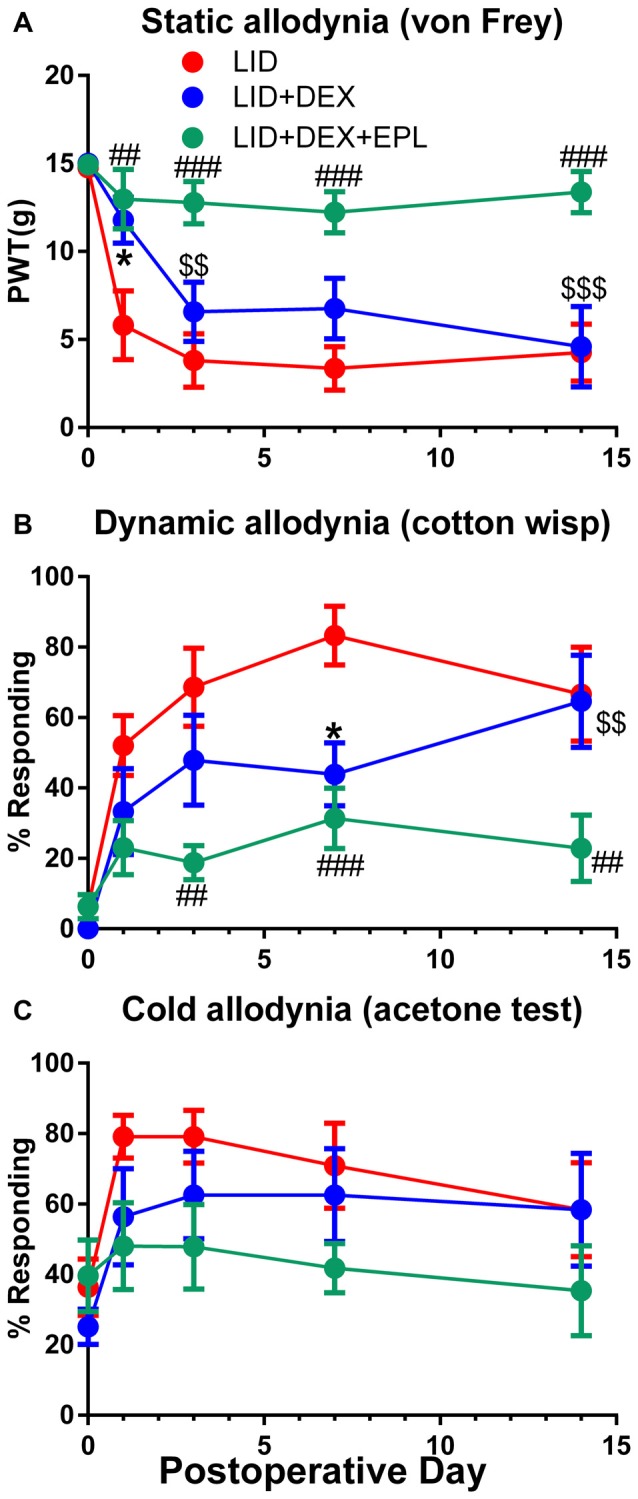
Eplerenone (EPL) enhances the analgesic effect of dexamethasone (DEX) after dorsal root ganglia (DRG) inflammation induced by local inflammation of the DRG (LID). Baseline behaviors were measured before surgery and the average baseline is plotted on postoperative day 0(POD 0). **(A)** Paw withdrawal threshold (PWT) to von Frey stimuli. **(B)** Dynamic allodynia (withdrawal response to light stroking with a cotton wisp). **(C)** Cold allodynia (withdrawal response to 50 μl of acetone applied on the paw). *p < 0.05 significant difference between LID and LID+DEX groups; ^##^*p* < 0.01; ^###^*p* < 0.001; significant difference between LID and LID+DEX+EPL groups; ^$$^*p* < 0.01; ^$$$^*p* < 0.001; significant difference between LID+DEX and LID+DEX+EPL groups at the indicated time points (two-way repeated measures ANOVA with Holm-Sidak posttest comparing each group with every other group, *P* = 0.0005, 0.0047 and 0.165 and *F*_(2,21)_ = 11.2, 7.0 and 2.0 for **(A–C)** respectively). Results shown combine equal numbers of both sexes (*N* = 4 males and 4 females per group).

Static, dynamic and cold allodynia behavioral assessments were performed for three experimental groups (LID, LID+DEX, LID+DEX+EPL). The DEX alone group showed significantly reduced mechanical pain behaviors compared to the LID group only at POD 1 (static) and POD 7 (dynamic) as shown in Figures 1A,B respectively. However, the effect was short and transient. After POD 7, the DEX effect declined and by POD 14 the behavior measures in the DEX group were not significantly different from those of the untreated LID group. Interestingly, EPL combined with DEX was more effective than DEX alone. The combination reduced mechanical pain behaviors more effectively, and this improvement was maintained up to POD 14. The behavioral effects were similar in males and females; both sexes were used for all experiments and data combined from males and females is presented throughout.

### EPL and DEX Combination Is Necessary for the Long Term Analgesia

**Figure 2:**
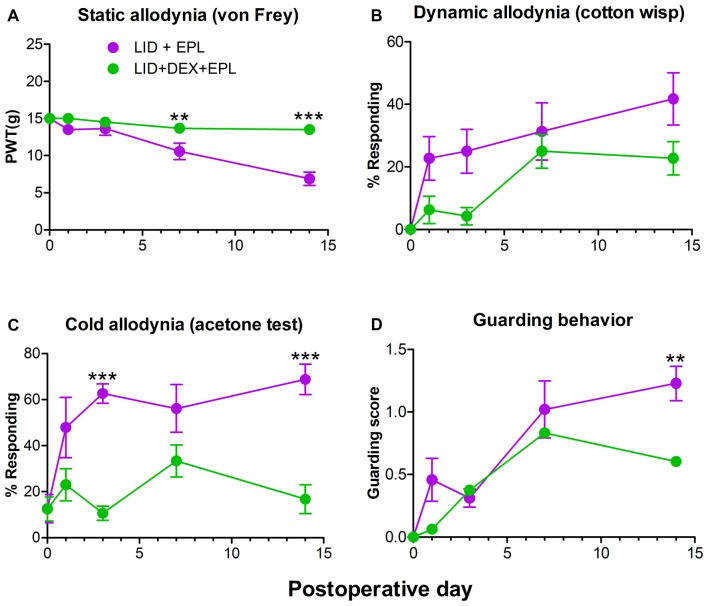
Combined EPL and DEX is needed for the long-term analgesia after local DRG inflammation (LID). Baseline behaviors were measured before surgery and the average baseline is plotted on POD 0. **(A)** PWT to von Frey stimuli. **(B)** Dynamic allodynia. **(C)** Cold allodynia. **(D)** Guarding behavior (a measure of spontaneous pain). **p < 0.01, ***p < 0.001; significant difference between LID+EPL and LID+DEX+EPL groups at the indicated time points (two-way repeated measures ANOVA with Holm-Sidak posttest comparing each group with every other group, *P* = 0.0009, 0.0478, <0.0001 and 0.0413 and *F*_(1,14)_ = 17.7, 4.7, 30.1 and 5.0 for **(A–D)** respectively. Results shown combine equal numbers of both sexes (*N* = 4 males and 4 females per group). The overall treatment effect between the two groups was significantly different using one-way ANOVA for all four measures, but did not always reach significance for individual time points.

Since we demonstrated that EPL improved the analgesic effect of DEX after local DRG inflammation (Figure 1), we next investigated if the combination of EPL and DEX was necessary for the pain reduction or EPL alone could do this. We conducted a second experiment comparing EPL alone to the combination of EPL + DEX. The combined treatment group provided stronger and sustained pain reduction compared to the EPL alone group. The most significant and distinct difference was noted at POD 14 (static, cold allodynia and guarding behavior) as indicated in Figure 2. In combination with the data from Figure 1 this demonstrates that the combined treatment can maintain the pain reduction much longer than either steroid alone (either DEX or EPL) up to POD 14. In this experiment, we also measured guarding behavior as a measure of spontaneous pain. At POD 14 the combination treatment dramatically reduced the spontaneous pain.

### The GR Is Widely Expressed in Both Neuronal and Non-neuronal Cells in the Normal DRG

**Figure 3:**
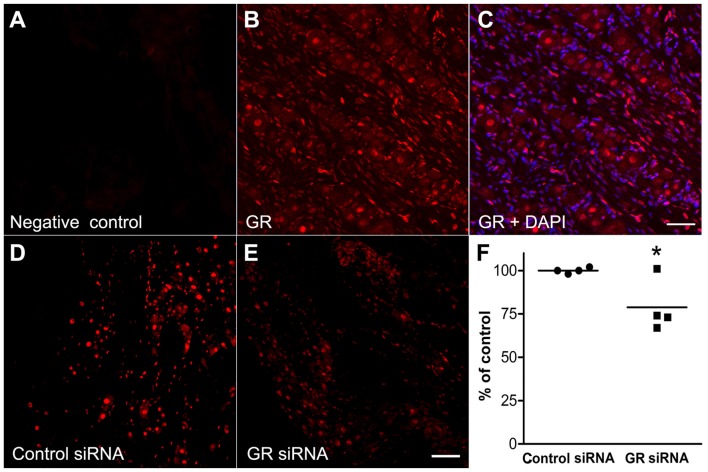
The glucocorticoid receptor (GR) is expressed in the nuclei of neuronal and non-neuronal cells in the normal DRG using the GR antibody M-20. **(A)** Example of GR negative control observed by omitting the primary GR antibody. **(B)** The GR which is visualized as the red color, **(C)** merged with DAPI, the fluorescent stain which visualizes nuclear DNA (blue). **(D)** GR immunoreactivity of GR in DRG injected with non-template control siRNA. **(E)** GR immunoreactivity of DRG injected with the anti-GR siRNA. Sections were obtained 4 days after siRNA injection into normal DRG. **(F)** Summary data of GR immunoreactivity intensity/area of DRGs injected with GR siRNA and non-targeting control siRNA. Each point represents average data from one animal, line indicates mean. *p < 0.05, significant difference between the groups (unpaired *t*-test, *p* = 0.03). The results shown combine equal number of both sexes (*n* = 2 males and 2 females per group, approximately 30–40 sections were used per group). Scale bar = 50 μm.

Using IHC in normal DRG sections, we found that the GR immunoreactivity was expressed in the nuclei of non-neuronal cells and especially in neurons within the DRG. Negative control sections were processed side by side with the normal DRG GR staining to check the specificity of the antibody by omitting the primary GR antibody and almost no signals were detected (Figure 3A). Nuclear localization was detected in the normal DRG (Figure 3B) using an antibody directed against a peptide mapping at the N-terminus of GR. 4’,6-diamidino-2-phenylindole (DAPI) the fluorescent stain which visualizes nuclear DNA (Tarnowski et al., 1991) is shown as blue and GR is visualized as red (Figure 3C). This predominantly nuclear GR staining pattern was observed in all the experiments/conditions in the study.

### Validation of the GR Antibody

The antibody used in this study demonstrated reduced staining after genetic or shRNA-mediated GR knockdown in brain neurons from mice and rats (Boyle et al., 2005, 2006; McKlveen et al., 2013). To further validate the antibody, non-targeting control siRNA (Figure 3D) was injected into the L4 and L5 DRGs in one group and GR siRNA (Figure 3E) in another group. Four days later L4 and L5 DRGs were collected from both groups (*n* = 4 rats in each group). Immunohistochemical staining showed that in animals injected with siRNA directed against GR, the GR immunoreactivity was significantly reduced compared to the non-targeting control siRNA. It appeared that ~25%–30% knockdown was observed in three animals and none in the fourth (Figure 3F).

The predominantly nuclear localization of the GR that we consistently observed in DRG neurons with the antibody used (M-20) is similar to that previously reported by some previous studies in neurons in various regions of mouse and rat brain (e.g., Murphy et al., 2002; Ostrander et al., 2003; Boyle et al., 2005, 2006; Furay et al., 2008; Rainville et al., 2017; Wei et al., 2017) but contrasts with a cytoplasmic location reported in other studies (e.g., Sarabdjitsingh et al., 2010; Jafari et al., 2012); with the latter study reporting nuclear localization only in cultured neurons but not hippocampal sections. These discrepancies highlight the need for validating antibodies within each study.

### GR^+^ Neurons Did not Differ From GR^−^ Neurons in Area

**Figure 4:**
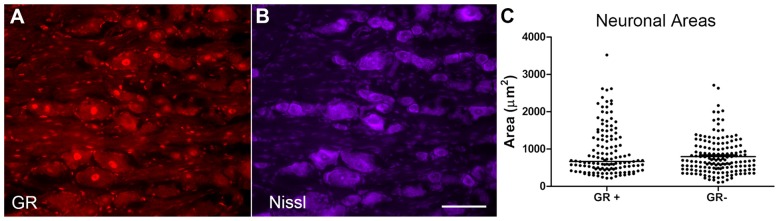
GR^+^ neurons did not differ from GR^−^ neurons in area. **(A)** GR (red). **(B)** Nissl stain (purple, neuronal stain). **(C)** Scatterplot showing cellular areas of GR positive (GR^+^) and GR negative (GR^−^) neurons; line indicates median. Areas of the GR^+^ neurons and GR^−^ neurons were not significantly different (Mann-Whitney test, *p* = 0.29). Scale bar = 50 μm. *N* = 2 male and 2 female animals per group.

The cross-sectional area of neurons with and without GR immunoreactivity was examined in normal DRG neurons, using the M-20 antibody against the GR (Figure 4A) and Nissl stain which allows for the visualization of all neurons (Figure 4B). Random sections were selected from four different animals, and random regions were selected from each section to calculate the cross-sectional area of GR^−^ neurons in that region vs. the GR^+^ neurons. Neurons across all size classes were observed to be GR^+^ (see individual data points in scatterplot, Figure 4C). The median of the GR^+^ neuronal area was not significantly different from the median GR^−^ neuronal area using Mann-Whitney analysis (*p* = 0.29).

### GR Is Also Expressed in a Subset of Non-neuronal, Especially Satellite Glia, Cells Within the Normal DRG

**Figure 5:**
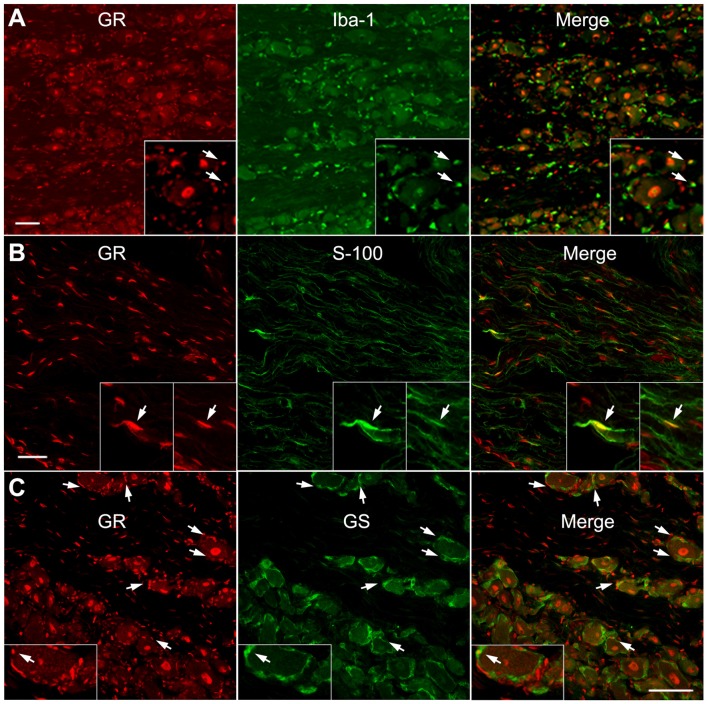
GR is co-expressed with some macrophage, satellite glia and Schwan cells within the normal DRG. Examples of double immunostaining of GR in sections from normal DRG with **(A)** Macrophage marker (Iba-1). **(B)** Schwann cell marker (S-100), observed in an axonal region. **(C)** Satellite glia marker (GS). Arrows indicate examples of double labeled cells. Similar results were obtained in 10–20 sections from each animal (*N* = 2 male and 2 female animals). Scale bar = 50 μm.

**Figure 6:**
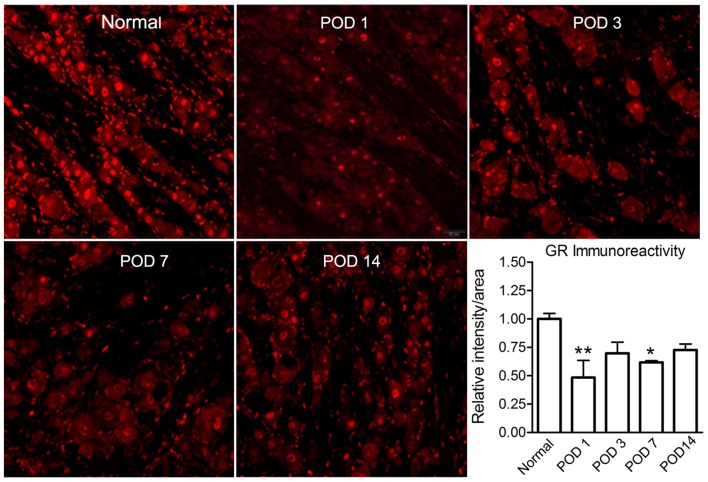
LID reduced the GR signal level. Examples of immunostaining of GR in sections from normal and inflamed DRG at different time points (POD 1, 3, 7 and 14). Scale bar = 50 μm. Summary data show GR intensity normalized to that seen in normal DRG measured side-by-side. *p < 0.05, **p < 0.01, significantly different from normal (one-way ANOVA with Dunnett’s posttest comparing each time point to normal DRG value; *p* = 0.02, *F*_(4,16)_ = 4.0). The results shown combine equal numbers of both sexes (*n* = 2 males and 2 females per group; 30–50 sections per animal).

As shown in Figures 3, 4, the most obvious GR staining was in the large neuronal nuclei. However, staining in smaller cell types could also be observed. As an initial examination of what other DRG cell types might also express GR, double immunohistochemical analysis was performed. This showed a subset of cells expressing either macrophage, Schwann cell (observed in axonal regions of the sections) or satellite glia markers (Figures 5A–C) in the normal DRG also expressed GR. Qualitatively, it appeared that satellite glia cells demonstrated the most GR co-localization of the three non-neuronal cell types examined, while the IbA-1-positive cells showed the least GR co-localization. However, none of the cell types examined displayed the degree of staining observed in neurons (Figures 3, 4, 6).

### Local Inflammation of the DRG Reduced the GR Immunoreactivity

We used immunohistochemical quantification to examine the time course of GR expression after LID. This analysis showed that the GR immunoreactivity was significantly reduced after inflammation (at POD 1 and 7; Figure 6). All values were normalized to the values in normal DRG measured in the same experiment. We also observed that, on POD14, a noticeable recovery of GR immunoreactivity could be especially observed in non-neuronal areas of the DRG (data not shown).

### Blocking MR With EPL Restored GR Immunoreactivity in the Inflamed DRG

**Figure 7:**
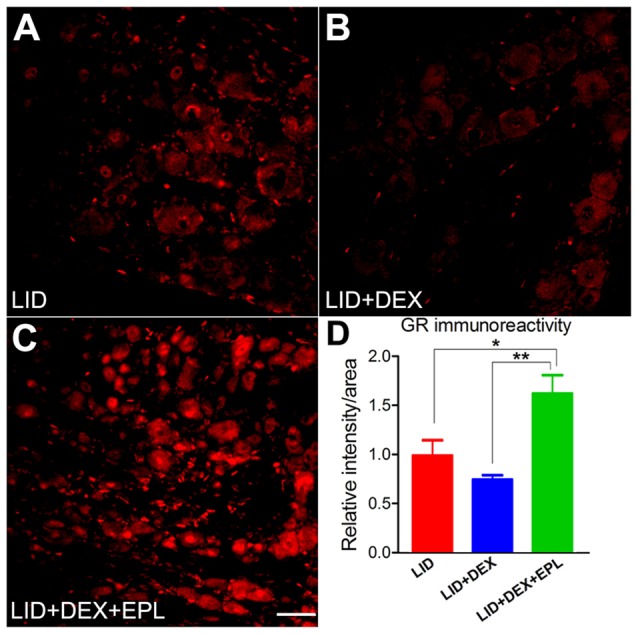
Blocking mineralocorticoid receptor (MR) with EPL restored GR signal level in the inflamed DRG. Examples of immunostaining of GR in sections from **(A)** Inflamed DRG, **(B)** Inflamed plus DEX, **(C)** Inflamed plus DEX+EPL, all at POD 14. **(D)** Summary data of GR immunoreactivity. All data are normalized to the LID group. *p < 0.05, **p < 0.01, significantly different from LID (one-way ANOVA with Tukey’s posttest comparing each group to every other group; *F*_(2,7)_ = 12.52, *p* = 0.0049). Observed in *N* = 2 males and 2 females per group, 30–40 sections per animal; samples taken from same animals as used in Figure 1. Scale bar = 50 μm.

Since the most striking behavioral effects of DEX + EPL were observed at POD 14, the GR immunoreactivity at POD 14 was compared between three groups: the LID group (Figure 7A), the LID+DEX group (Figure 7B), and the LID+DEX+EPL group (Figure 7C). At POD 14, the GR immunoreactivity in the DEX group was not significantly different from the LID group. This correlates with the behavior pain assessment at POD 14, where DEX group was not significantly different from the LID in any of the pain assays. However, when EPL was added to the DEX treatment (Figure 7C), it surprisingly increased the GR immunoreactivity compared to the DEX alone treatment. The GR immunoreactivity was significantly higher in the combined treatment than in the LID group, and also higher than the DEX alone group (Figure 7D).

### Blocking MR With EPL Reduced GFAP Levels in the Inflamed DRG

**Figure 8:**
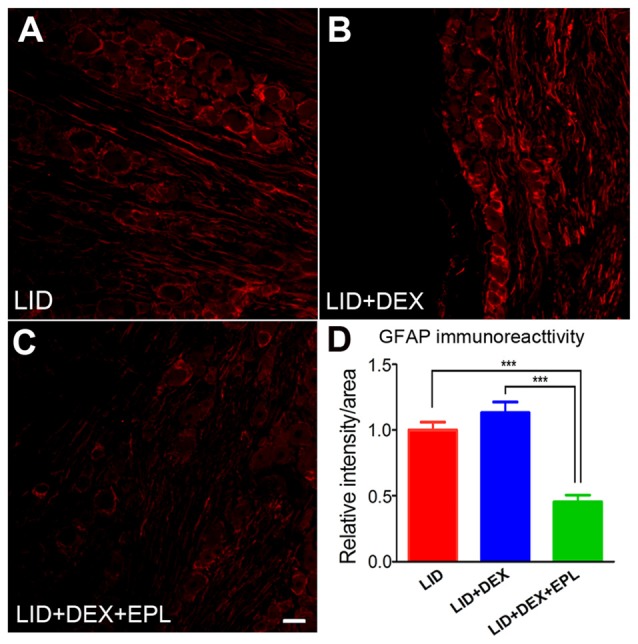
Blocking MR with EPL reduced glial fibrillary acid protein (GFAP) levels in the inflamed DRG. Examples of immunostaining of GFAP in sections from **(A)** Inflamed DRG, **(B)** Inflamed plus DEX, **(C)** Inflamed plus DEX+EPL, all at POD 14. **(D)** Summary data of GFAP immunoreactivity. All data are normalized to the LID group. ***p < 0.0001, significantly different from LID (one-way ANOVA with Tukey’s posttest comparing each group to every other group; *F*_(2,9)_ = 30.20, *p* = 0.0001). Observed in *N* = 2 males and 2 females per group, 20 sections per animal; samples taken from same animals as used in Figure 1. Scale bar = 50 μm.

We examined GFAP, a marker for satellite glia activation and neuroinflammation in the DRG (Hanani, 2005). The GFAP immunoreactivity was compared between three groups: the LID group (Figure 8A), the LID+DEX group (Figure 8B), and the LID+DEX+EPL group (Figure 8C) to determine whether the increased GR signal was accompanied by reduced inflammation on POD 14. The GFAP immunoreactivity at POD 14 in the DEX group was not significantly different from the LID group. However, when EPL was added to the DEX treatment (Figure 8C), it significantly lowered the GFAP immunoreactivity compared to the DEX alone treatment and the LID group (Figure 8D).

### The Level of mRNA for GR but Not MR Was Altered in the Inflamed DRG

**Figure 9:**
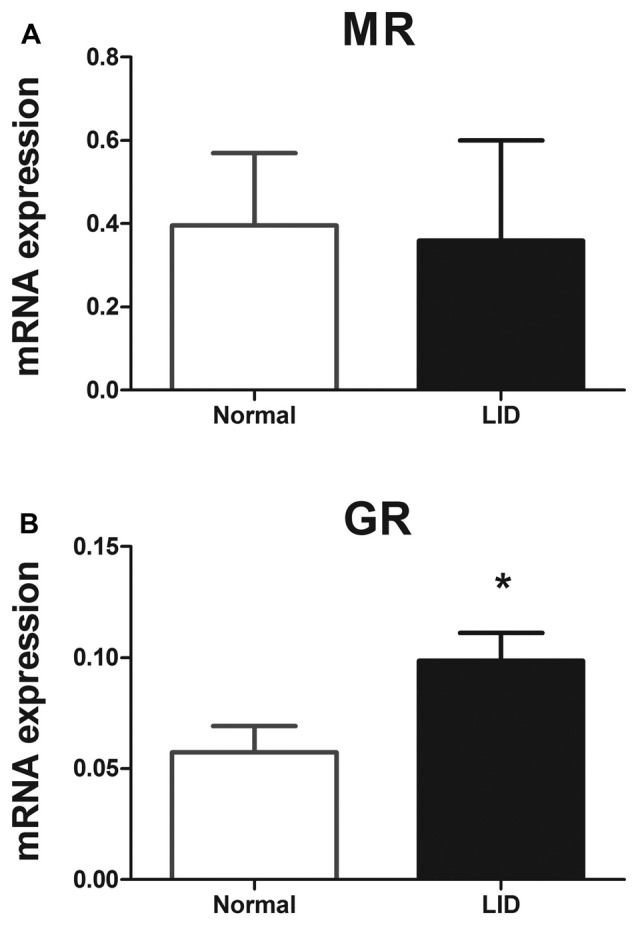
The level of mRNAs for GR but not MR was altered in the inflamed DRG at POD 1. mRNA gene expression levels of the **(A)** MR and **(B)** GR. mRNA expression was normalized to the housekeeping gene Hypoxanthine Phosphoribosyl transferase (HPRT). *p < 0.05, significantly different from normal (unpaired *t*-test; *p* = 0.90 for MR and 0.043 for GR). Both sexes were used (four males and four females per each group).

GR and MR signaling are regulated at different levels including the amount of endogenous corticosteroid available locally, the interacting proteins in the cytoplasm and in the nucleus such as transcriptional co-factors, and posttranslational modifications such as phosphorylation, sumoylation, ubiquitination and nitrosylation (Dejager et al., 2014; Faresse, 2014). To look for possible regulation at the mRNA level, we used quantitative PCR, and found that MR gene expression levels 1 day after DRG inflammation did not show any significant difference compared to normal DRG, while GR gene expression level showed a 1.7-fold upregulation (Figure 9).

### Local Inflammation of the DRG Reduced the GR Expression in the DRG

**Figure 10:**
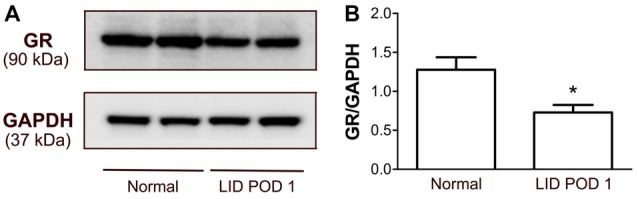
Detection of GR protein expression in the DRG of normal and LID-treated rats using Western blot analysis. **(A)** Examples of observed bands. **(B)** Quantification of GR protein showed reduction of the GR protein in the DRG 1 day after LID compared to normal DRGs from rats. *p < 0.05, significant difference between the groups (unpaired *t*-test, *p* = 0.015). The results shown combine equal number of both sexes (*n* = 3 males and 3 females per group).

Because the largest decrease in GR immunoreactivity after DRG inflammation was observed on POD 1, a time point when mRNA levels increased, we also used Western blot analyses to examine GR protein levels at this time point. The Western blot of DRG proteins identified a single GR-specific protein band at 90 kDa. The optical density of the GR bands extracted from the DRG decreased significantly after 1 day of the LID to ~57% of the level observed in normal DRGs (Figure 10), consistent with the 48% reduction observed in the immunohistochemical experiment (Figure 6).

## Discussion

Since inflammation is involved in most back pain conditions, anti-inflammatory drugs such as epidural steroid injections are commonly used to relieve pain symptoms. Activation of satellite glia cells, infiltration of macrophages, increase in pro-inflammatory cytokines and activation of inflammatory signaling pathways can be observed as an inflammatory response in low back pain models. (Kawakami et al., 1996; Xie et al., 2006; Amaya et al., 2009; Otoshi et al., 2010; Huang et al., 2011; de Souza Grava et al., 2012). Steroids injected clinically for back pain target the GR for its desired anti-inflammatory effects. *In vivo*, the synthetic glucocorticoid we used in this study, DEX, is more selective for the GR compared to the MR, but still has considerable potency in activating the MR (GR EC_50_ is 0.561 nM, MR EC_50_ is 5.09 nM, a ratio of 0.11). Other steroids clinically used for epidural injection also activate the MR to some degree, e.g., in order of increasing GR specificity, 6-α-methylprednisolone (ratio of 1.27), triamcinolone (0.12), and betamethasone (ratio of 0.02; Grossmann et al., 2004). These values are from *in vitro* measures of GR/MR activation of reporter gene transcription. However, GR exerts its anti-inflammatory effects not only by directly activating gene transcription, but especially by binding to inflammatory transcription factors such as NF-κB and AP-1 and repressing their activity, thus downregulating the transcription of inflammatory genes that themselves may lack the regulatory DNA sequences recognized by the GR (Smoak and Cidlowski, 2004). Conversely, the MR may exert pro-inflammatory effects in part by upregulating these same inflammatory transcription factors (Neves et al., 2005; Li et al., 2007; Leroy et al., 2009). Our initial hypothesis was that steroids that can activate both the MR and the GR might be less effective at relieving pain in the DRG inflammation model because the pro-inflammatory MR effects could compete with the anti-inflammatory GR effects via these transrepression mechanisms, although it should be noted that potency for gene activation (as measured in the reporter gene experiments) does not always precisely correlate with potency for transrepression (Lu et al., 2006; Dirks et al., 2008; Joanny et al., 2012).

The behavioral experiments in this study were consistent with this simple hypothesis. We found that LID with zymosan/IFA induced pain behaviors starting from POD 1 up to POD 14. Pain behaviors have previously been shown to continue for up to 8 weeks (Xie et al., 2012b). We found that combining the MR antagonist EPL with the clinically used epidural steroid DEX made DEX more effective in reducing pain behaviors. In particular, at 2 weeks, the pain scores with DEX alone did not differ from scores in untreated animals, while pain scores in DEX plus EPL treated animals were close to the baseline. The pain improvement at POD 14 in the combined group compared to the DEX alone group correlated with the increased GR levels and decreased neuroinflammation (GFAP) observed in the DEX + EPL group. These results are also consistent with our previous study, where combining EPL with a different steroid, 6-α methylprednisolone, enhanced its effectiveness in reducing pain behaviors after local DRG inflammation (Ye et al., 2014). Another study reported similar findings using a different back pain model (chronic compression of the DRG). They documented that intrathecal spironolactone (MR antagonist) or GR agonist (DEX) reduced radicular pain behaviors, with the combination giving synergistic effects. In addition, a GR antagonist (mifepristone) exacerbated the pain behaviors (Gu et al., 2011). These results suggest that blocking the MR can be beneficial in reducing pain behaviors when an MR antagonist is combined with the currently used steroids.

Our immunohistochemical experiments demonstrated that the GR was present most notably in neurons and also in some non-neuronal cells in the DRG, and was primarily observed in the nucleus with the antibody we used. Expression of the GR in the rodent DRG has been previously described (DeLeón et al., 1994; Lerch et al., 2017; Rijsdijk et al., 2017; Li et al., 2018), though there are some discrepancies between studies about the details of receptor distribution. GR expression in satellite glial cells has also been previously observed (Rijsdijk et al., 2017); as in our study, it appeared only a subset was GR-positive. Our previous study showed that many DRG neurons also express the MR (Dong et al., 2012), and others have directly demonstrated co-localization of GR and MR within DRG neurons (Li et al., 2018), as well as within GR-rich regions of the brain such as hippocampus (Gomez-Sanchez and Gomez-Sanchez, 2014). Interestingly, the GR levels were approximately 15 times higher in DRG than in the hippocampus (Lerch et al., 2017). Therefore, interactions between these two receptors may have unique features in the DRG that are relevant to the action of epidural steroid injections.

The immunohistochemical experiments showed that the GR immunoreactivity is down-regulated by DRG inflammation starting within 1 day. This GR immunoreactivity downregulation after 1 day of inflammation was confirmed by Western Blot analysis. On day 14, when EPL supplementation was most effective behaviorally, this GR downregulation was reversed by EPL supplementation. Our primary hypothesis of GR and MR simply having competing transrepression effects did not predict that inflammation would downregulate the GR immunoreactivity/expression, or that EPL when combined with DEX might enhance this GR reduction compared to DEX alone. This suggests additional and more complex mechanisms for the observed behavioral effects of DEX and EPL. The GR downregulation observed after DRG inflammation might account for the reported lack of efficacy in patients treated with steroid injections for low back pain management. Downregulation of GR after inflammation has been reported in other systems, based on multiple mechanisms. Increased endogenous corticosteroids can downregulate GR at both the RNA and protein levels (homologous downregulation; Dejager et al., 2014); we have previously provided evidence against the idea that DRG inflammation GR elevates corticosteroids systemically (Dong et al., 2012), but local corticosteroid increases would be consistent with the 1.4-fold upregulation of hydroxysteroid 11-beta dehydrogenase 1 after DRG inflammation observed in our previous microarray study (Strong et al., 2012). Arguing against this particular mechanism is our observation that GR mRNA was upregulated, not downregulated, by DRG inflammation. Other possible mechanisms for the observed GR downregulation are suggested by studies showing that, although GR can downregulate inflammatory cytokines, inflammatory cytokines can also downregulate GR via multiple direct and indirect mechanisms, including some posttranslational mechanisms (Dejager et al., 2014). These mechanisms are cytokine- and cell-type-specific. The rescue of GR levels by the MR antagonist EPL could therefore in part be due to reduction of inflammation by EPL, though the relatively modest effects of EPL alone on behavior at day 14 suggests this is not the only mechanism. Another mechanism by which GR and MR may interact specifically includes the formation of MR/GR heterodimers (including in neurons; Nishi et al., 2004), in which the MR may have a dominant negative effect (Planey et al., 2002). Heterodimers may have different transcriptional activities than the MR or GR homodimers. MR and GR may also interact via competition for ligands, regulatory proteins, and DNA response elements, and through regulation of each other’s transcription (Gomez-Sanchez and Gomez-Sanchez, 2014).

In the GR immunohistochemical time course experiment, we noticed that at POD 1, there was still a considerable amount of neuronal GR vs. non-neuronal GR compared to POD 14 where there was more non-neuronal GR vs. neuronal GR. Also, we noticed that DEX alone was more effective in the early time points vs. POD 14 where the DEX behavioral effect was negligible. This suggested that neuronal GR rather than total or non-neuronal GR immunoreactivity can be correlated to the behavioral effectiveness of the DEX. The importance of neurons as a site of steroid action is also supported by our previous studies showing that EPL or GR agonists applied *in vitro* reduced hyperexcitability of sensory neurons isolated from inflamed DRGs (Dong et al., 2012; Ye et al., 2014). A recent study using a paw inflammation model provided evidence for a key role of sensory neurons in mediating anti-nociceptive responses to systemic glucocorticoids and MR antagonists (Li et al., 2018). However, in that study, observed behavioral effects were rapid and attributed to plasma membrane receptors mediating nongenomic effects. Because in our study, we did not observe plasma membrane labeling, but rather primarily nuclear staining, we are not able to make inferences about possible roles of nongenomic steroid effects.

A limitation of the current study is that only one measure of inflammation was examined. It would be of interest to examine the effects of EPL supplementation on the cytokine profile. In addition, it would be of greater clinical relevance to apply the steroids after the pain was established, which is technically more difficult to do in this rat model.

Overall, our results suggest that blocking the undesirable effects of MR activation by combining MR blockers with clinically used epidural steroids can improve the effectiveness of the currently used steroids, not only through antagonizing the deleterious effects of MR activation, but also due to the enhancement of the GR immunoreactivity which may allow for more of the desired anti-inflammatory effects to occur. This can open a new window for improved treatment of low back pain relief. EPL is already approved for other clinical conditions such as treatment of hypertension and heart failure (Kolkhof and Bärfacker, 2017). This facilitates trials testing its efficacy in potentiating epidural steroid injection treatments. The highly selective GR agonist fluticasone (Austin et al., 2002) can be theoretically considered as an ideal drug to be used as epidural steroid injections in managing low back pain, and was highly effective in reducing pain behaviors in our DRG inflammation model (Ye et al., 2014). However, fluticasone is not approved for the use as an epidural steroid injection. It is mainly used in the form of nasal spray and aerosol for treatment of allergies and asthma. In addition, since MR may be activated by DRG inflammation, blocking the MR might potentiate effects of even a highly GR-selective steroid.

Inflammation is a component of many different models of both inflammatory and neuropathic pain, not just low back pain models. Therefore, it will be interesting to determine the effects of MR antagonists in other pathological pain conditions such as arthritis and painful joint inflammation that are currently treated with GR agonists, which also activate the MR *in vitro*.

## Author Contributions

SI conducted the experiments, analyzed the data, designed the experiments and drafted the manuscript. WX conducted the experiments. JS analyzed the data, designed the experiments and edited the manuscript. RT conducted the experiments and analyzed the data. TB analyzed the data and edited the manuscript. J-MZ designed the experiments and edited the manuscript.

## Conflict of Interest Statement

The authors declare that the research was conducted in the absence of any commercial or financial relationships that could be construed as a potential conflict of interest.
